# Primary Chronic Osteomyelitis of the Jaws in Children: An Update on Pathophysiology, Radiological Findings, Treatment Strategies, and Prospective Analysis of Two Cases

**DOI:** 10.1155/2015/152717

**Published:** 2015-09-07

**Authors:** Caroline Berglund, Karin Ekströmer, Jahan Abtahi

**Affiliations:** ^1^Department of Oral & Maxillofacial Surgery, Linköping University Hospital, 581 85 Linköping, Sweden; ^2^Department of Radiology, Mälarsjukhuset Eskilstuna Hospital, Sweden

## Abstract

*Objective.* Primary chronic osteomyelitis (PCO) of the jaws in children is associated with pain, trismus, and swelling. In children, temporomandibular joint involvement is rare and few studies have been published due to the relatively low incidence. This paper presents two cases of mandibular PCO in children with the involvement of the collum mandibulae. In addition, a review of the literature regarding demographic data, histological, radiological, and laboratory findings, and treatment strategies of PCO was also performed. *Material and Methods.* Prospective analyses of two PCO cases. A PubMed search was used and the articles were sorted according to their corresponding key area of focus. *Results.* Review of the literature revealed twenty-four cases of PCO with two cases of mandibular condyle involvement. The mean age was 18 years; the male to female ratio was 1 : 3. Most of the patients were treated with anti-inflammatory drugs in combination with decortication. Clinical recurrence was seen in 7 cases. *Conclusion.* A combination of anti-inflammatory drugs and surgical intervention appears to be the first choice of treatment. However, surgical removal of necrotic tissue adjacent to collum mandibulae has its limitations in children. Further investigations are of utmost importance in order to increase our knowledge and understanding of this disease.

## 1. Introduction

Osteomyelitis has been defined as an inflammatory state of cortical and cancellous bone and the most affected sites are the long bones of extremities [1]. Osteomyelitis can be either acute or chronic. The term chronic osteomyelitis is used for primary or secondary cases with duration of more than 4 weeks from the onset of symptoms [2]. Secondary chronic osteomyelitis of the jaw (SCO) is usually caused by bacterial infection of dental origin (pulpal disease, posttooth extraction, or foreign bodies) and is much more common than primary chronic osteomyelitis [3]. Primary chronic osteomyelitis (PCO) is a nonbacterial chronic inflammatory disease of unknown etiology, which can also be associated with other conditions, including autoimmune diseases and syndromes such as “SAPHO (Synovitis, Acne, Pustulosis, Hyperostosis, and Osteitis) syndrome,” Majeed syndrome, and cherubism [1]. Hematogenous spread of inflammation has also been mentioned in the literature, especially in osteomyelitis of the long bones of infants and children [4].

Primary chronic osteomyelitis of the jaw has been reported in children of both sexes with a peak onset between 10 and 20 years [5]. In the maxillofacial region, the most affected site is the mandible [2, 6, 7].

The bacterial contamination of bone tissue is best determined by bone biopsy under radiographic guide [8]. The most frequently bacteria associated with osteomyelitis are* Staphylococcus aureus*, Gram negatives (*Pseudomonas aeruginosa*), and anaerobe bacteria (*Bacteroides fragilis*) [8].

Radiographic and histological findings are characteristic but not pathognomonic of PCO, which make a diagnostic challenge for the maxillofacial surgeon. Several imaging modalities have been used in patients with PCO of the jaw, including panoramic radiograph, CT scan, cone beam computed tomography (CBCT) scan, MRI, and bone scintigraphy [9–12]. Radiological examination reveals the presence of radiolucent areas combined with progressive osteosclerosis, lytic lesions (radiolucency), and laminations of periosteal new bone [13]. The occurrence of bone sequestration is an uncommon feature in patients with PCO [3, 14]. Histological findings show diffuse medullary fibrosis in combination with subperiosteal reactive bone formation and chronic inflammatory infiltration [14].

Panoramic images and intraoral images provide an overview of the jaw and teeth health status and are also used to show any possible dental infection. Computed tomography and magnetic resonance imaging are increasingly used in evaluating osseous pathology of the maxillofacial skeleton [9]. For concerns for increased radiation exposure in children, CBCT can be used with advantage [15]. Bone scintigraphy is highly sensitive to bone abnormalities and is a useful method especially in patients with suspected inflammatory disease or malignant tumors [16, 17]. However, this method is often used for early diagnosis of any inflammatory process in the whole skeleton [18].

Treatment of primary chronic osteomyelitis of the jaw includes anti-inflammatory drug therapy such as corticosteroids in combination with surgical removal of inflammatory or necrotic bone tissue [19]. Nonsteroidal anti-inflammatory drugs (NSAID) are beneficial and considerably improve the patient's quality of life [14]. Antibiotics have been given most of preventive reasons and hyperbaric oxygen therapy has been used with varying results [20].

We present two cases of PCO in children with involvement of corpus/angulus mandibulae and condyle. In addition, a literature review was performed regarding clinical, radiological, laboratory, and histological findings. Treatment strategies and various aspects of surgical intervention have also been discussed in this paper.

## 2. Materials and Methods

A PubMed search was made using the MeSH term “primary chronic osteomyelitis of the jaw.” Twenty-six studies were found. Sixteen studies had reported jaw involvement as a part of this disease. Cases with purely mandibular and midface involvement were distinguished from cases associated with other syndromes. Ten studies with purely facial skeleton involvement were included, consisting of 2 reviews and 8 case reports. The present paper is authored as a narrative review contribution. The articles were sorted according to their corresponding key area of focus. Parent consent has been taken prior to approval from ethical committee.

## 3. Terminology and Definition

An overview of the literature on osteomyelitis of the jaw reveals a wide variety of proposed classifications based on different aspects such as clinical course, radiological features, pathogenesis, and etiology. However, most agree that osteomyelitis can be classified as acute or chronic. Acute osteomyelitis differs from chronic osteomyelitis, which has duration of four weeks after the onset of clinical symptoms [21, 22]. Many authors advocate that chronic osteomyelitis involving the jawbone can be further divided into two major categories: suppurative and nonsuppurative forms [23–26]. The main cause of chronic suppurative osteomyelitis of the jaws is odontogenic infections, which might occur as a complication of dental extractions, maxillofacial trauma, or irradiation of the facial skeleton [27–29]. The clinical findings of this disease were characterized by the presence of pus, fistula, and sequestration [28–30]. Odontogenic microorganisms such as* S. aureus*,* S. epidermidis*, and* Actinomyces* usually contribute to the pathogenesis of osteomyelitis of the jaws [31].

The term nonsuppurative osteomyelitis is characterized by the absence of the formation of pus, fistula, and sequestration [31–36]. These forms of osteomyelitis of the jaw include osteoradionecrosis, bisphosphonate-related osteonecrosis of the jaws (BRONJ), chronic recurrent multifocal osteomyelitis of children, and chronic sclerosing osteomyelitis [31–36]. Diffuse Sclerosing Osteomyelitis (DSO) is a radiographic term that has been used to describe the radiographic pattern associated with both PCO and CSO [37]. Chronic recurrent multifocal osteomyelitis (CRMO) is a nonautoimmune disorder that mostly affects children and is characterized by periods of exacerbations and remissions over many years [32, 38, 39]. It causes periodic bone pain, fever, and the appearance of multiple bone lesions that can occur in any skeletal site. SAPHO syndrome is the adult version of CRMO, which is associated with synovitis, acne, pustulosis, hyperostosis, and osteitis [40].

Primary Chronic Osteomyelitis (PCO) is a nonodontogenic and nonsuppurative chronic inflammatory condition of unknown origin [30]. Several authors have pointed out a possible association between primary chronic osteomyelitis of the jaw and other syndromes, such as CRMO, DSO, and SAPHO [41–43].

### 3.1. Clinical Manifestations and Differential Diagnoses

PCO is reported in children and adults of both sexes with two peak incidences between 10 and 20 years of age and above 50 [2]. The presenting symptoms are usually those of mandibular pain, swelling in lower jaw region, paresthesia over affected area and/or lower lip (Vincent's symptom), and limitation in mouth opening (trismus) [2]. Clinical findings also include enlargement of regional lymph nodes [3]. The course of disease is usually chronic (>4 weeks), intermittent (each episode lasting from a few days to several weeks with silent periods between them), and often refractory to antibiotics [1]. No predisposing factors such as traumatic injuries, radiation, or chemical substances exposure have been seen in these patients [2].

The differential diagnosis is broad and includes infectious osteomyelitis, fibrous dysplasia, ossifying fibroma, sialadenitis, parotitis, and malignancy such as osteosarcoma [1]. This condition may also accompany extragnathic symptoms such as those in SAPHO syndrome including synovitis, acne, pustulosis, hyperostosis, and osteitis [1–3].

### 3.2. Pathophysiology/Etiology

The etiology of PCO remains uncertain and theories include bacterial infection, autoimmune response, and vascular insufficiency due to localized end arteries, especially in the mandible [32, 37]. There is increasing evidence for the theory that PCO is genetically driven [44–46]. The importance of autoinflammatory response (interleukin-10, IL-10) in the development of PCO has been discussed by several authors [1, 44–46]. An impaired gene expression of IL-10 with subsequent disruption of the anti-inflammatory balance might explain part of the clinical presentation of PCO. This view is supported by the animal models with genetic defects [47–49]. No bacterial contamination has so far been demonstrated in a patient with PCO [31].

### 3.3. Laboratory Findings

Laboratory results are nonspecific and usually limited to mild increase in sedimentation rate and C-reactive protein level [6]. Malignancy and bacterial osteomyelitis are often included in the differential diagnosis of the PCO lesions; therefore it is vital to retrieve bone biopsy early, in order to diagnose and manage these patients [38, 39]. Histological examination of samples obtained by biopsy may show chronic, nonspecific inflammatory lesion with infiltration of plasma cells and variable presence of neutrophilic granulocytes, lymphocytes, and macrophages [2, 31]. Medullary fibrosis and endosteal bone apposition with pagetoid (irregular of reversal lines) reaction are more prominent in advanced disease and elderly patients, while bone resorption and subperiosteal bone formation are more noted in the early stage of disease and in younger patients [2]. Extraoral sampling or utilizing polymerase chain reaction (PCR) analysis may yield more reassuring results, but further studies are needed [31].

### 3.4. Radiological Analysis and Findings

Several imaging modalities have been used for diagnostic of PCO, including panoramic radiograph, CT scan, CBCT scan, MRI, and skeletal scintigraphy [9–12]. Radiological examination reveals the presence of radiolucent areas, bony sequester, and laminations of periosteal new bone [13].

Panoramic radiograph is an extraoral radiographic technique widely used by many dentists and oral and maxillofacial surgeons. This view is commonly used to assess an abnormal bone model, an expansion or asymmetry of skeleton, loss of the normal trabecular architecture, absence of the cortical outline of the mandibular canal, temporomandibular joints, and maxillary sinuses. Although panoramic images provide an excellent overview of the facial skeleton and teeth, the reliability of measurements has been under criticism. Panoramic images can vary widely as they depend on both position of the patient and the operator [50].

Computed tomography is increasingly used in evaluating osseous pathology in the maxillofacial skeleton. It is an excellent tool for assessing the relative distribution of cortical and cancellous bone and can assist in identifying appropriate location for bone biopsy [50, 51]. The typical appearance of osteomyelitis of the jaw on CT scan images is that the axial slices can reveal a thickening of the bone with strong periosteal reaction. Cone beam computed tomography (CBCT) technique can be used to ensure a low radiation dose, especially in children [15].

In several maxillofacial departments, MRI is an established imaging modality to assess osteomyelitis of the jaw. MRI examination generates no ionizing radiation [11, 12]. It shows both bone reactions and possibly involvement of the adjacent soft tissues [11, 12]. MRI may even show the extent of the lesions before reactions are seen in X-ray images. MRI T_1_ weighted images are the best as the pathologic process creates low signal intensity in the normally bright signal of fat contained in the marrow. However, MRI examination and waiting time are longer for the patient, and MRIs are not as commonly available in public health care.

Bone scintigraphy is highly sensitive to bone abnormalities and is a useful method especially in patients with suspected or known inflammatory disease or malignant tumors [16, 17]. This radiological method is generally used in combination with other diagnostic imaging techniques, due to low specificity and low spatial resolution. A bone scan image provides a functional display of skeletal activity and is rapidly positive within the first 24–48 hours after the onset of symptoms [52, 53]. As functional change in bone occurs earlier than structural change, the bone scan image gives us a hint where the activity is increased and where the next onset of symptoms will occur.

## 4. Treatment

Initially, antibiotics are often used empirically to prevent any bacterial invasion in acute and secondary chronic osteomyelitis. However, chronic infection remains an unproven theory for primary chronic osteomyelitis. Nonsteroidal anti-inflammatory drugs and corticosteroids are reported to have beneficial effects in reducing symptoms such as extraoral swelling and trismus [8, 30].

The importance of decortication and removal of necrotic bone tissue in primary chronic osteomyelitis of the mandible have been discussed by several authors [2, 14, 37]. Few cases have been reported regarding the treatment of primary chronic osteomyelitis of the mandibular condyle in children. Surgical intervention of condyle is a possible method in adults. However, in children, this may cause a disturbance of the mandible growth [54].

It has been suggested that PCO is genetically driven and this condition acts on the basis of an autoinflammatory response [44–46]. Nonsteroidal anti-inflammatory drugs and corticosteroids were also used as first-line options in other autoinflammatory bone diseases including familial chronic multifocal osteomyelitis which is also referred to as Majeed syndrome, sporadic chronic recurrent multifocal osteomyelitis (CRMO), and Synovitis, Acne, Pustulosis, Hyperostosis, and Osteitis (SAPHO) syndrome [55–57]. Second-line options include methotrexate, anti-TNF, sulfasalazine, or bisphosphonate [58–61]. Bisphosphonate therapy in chronic noninfectious inflammatory bone lesions is based on its anti-inflammatory and antiresorptive properties [58–60]. However, because of the side effects of long-term treatment in children, these drugs should be used in severe cases and in cases resistant to conventional therapy [62, 63].

The use of hyperbaric oxygen therapy (HBOT) as an adjunct to anti-inflammatory and surgical treatment has been advocated in children with osteomyelitis [20]. Higher levels of available oxygen cause induction of capillary formation and increased activity of leukocytes [64]. The HBOT also stimulates the release of growth factors and stem cells, which promote healing [64]. However, in the literature, studies with this therapy have been reported, with various results [20, 64].

## 5. Report of Cases

### 5.1. Case  1

**Figure 1:**
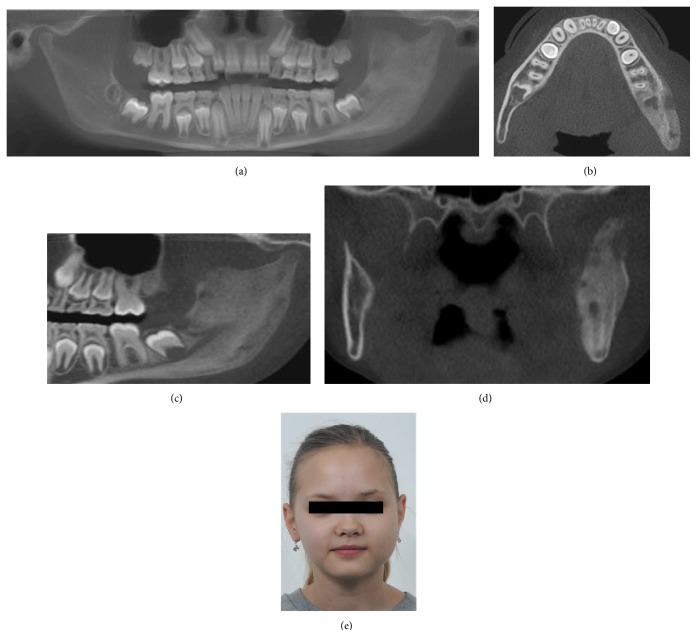
Preoperative CBCT and clinical image of patient case 1 with PCO. (a) Panoramic CBCT; (b, c, and d) axial, sagittal, and coronal sections of sclerotic mandible with new bone formation and lytic lesion at left posterior site; and (e) patient photo with swelling of the left side of the face.

A 10-year-old girl was referred to the Department of Otorhinolaryngology and Oral and Maxillofacial Surgery, Eskilstuna Hospital, Sweden, for evaluation of facial chronic swelling at the left side. According to the parents, the whole family was on holiday at the Mediterranean coast 8 months before they visited our department. A few weeks after patient's return to Sweden, the patient experienced swelling of regional lymph nodes at the left side of the neck. No further investigation was done. Two months later, the patient had noticed a slight swelling at the left side of cheek (Figure 1(e)) and showed restricted mouth opening. The general practitioner suspected TMJ-like symptoms and referred the patient to Dental Oral Rehabilitation Center. Dental examination did not give any hint of inflammatory dental foci. There was no previous history of viral or bacterial infection. Periapical radiographs showed no significant pathology that could explain the patient's symptoms. Magnetic resonance imaging (MRI) showed inflammation adjacent to the left side of ramus mandible.

**Figure 2:**
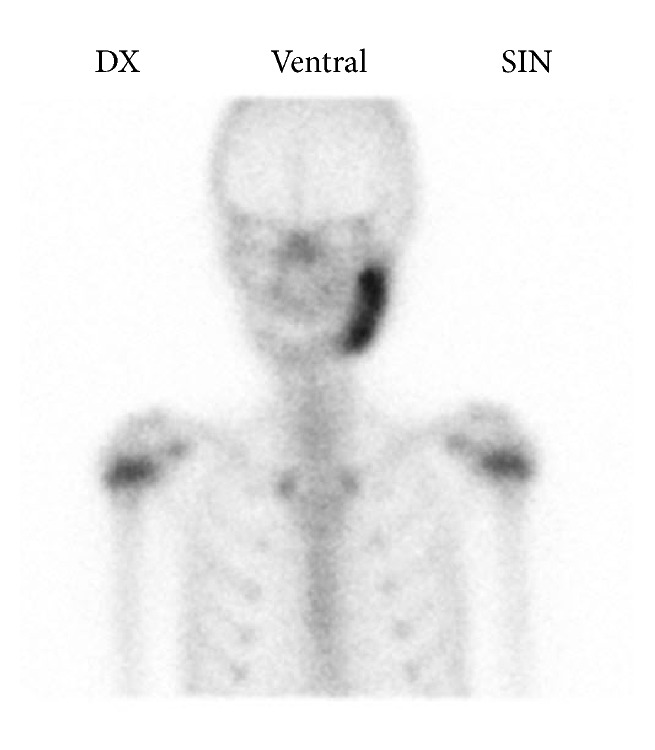
Bone scintigraphy of patient case 1 shows increased uptake of tracer at the left side of the mandible.

On the basis of these findings the patient was then referred to the Department of Otorhinolaryngology and Oral and Maxillofacial Surgery for further investigation. Panoramic CBCT demonstrated loss of the normal trabecular pattern with radiopacity within ramus/angulus mandibulae and incomplete development of germs of the third molar at the left side (Figure 1(a)). Moreover, sectional images of CBCT showed typical periosteal reaction, lytic lesions, and new-bone formation at the left side, from ramus mandibulae to incisura/collum mandible (Figures 1(b)–1(d)). Bone scintigraphy showed increased tracer uptake only at the left side of the mandible (Figure 2).

**Figure 3:**
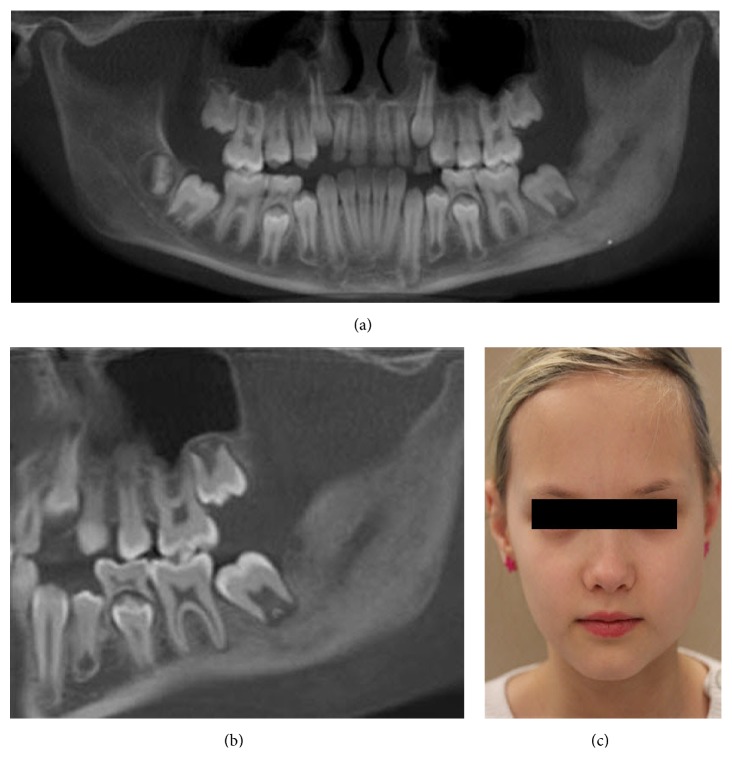
Six-month postoperative control CBCT and clinical image of patient case 1 with PCO. (a) Panoramic CBCT; (b) sagittal section of sclerotic mandible with no lytic lesion; and (c) patient photo, with reduced facial swelling at the left side.

Although the diagnosis of osteomyelitis was considered probable, the possibility of a tumorous lesion could not be excluded. The patient was submitted to diagnostic bone biopsy. Under general anesthesia, several bone biopsies were then taken for histological and microbiological examination. The histological findings were consistent with chronic inflammation. No signs of malignancy were seen. Bacterial cultures showed no growth of organism, such as mycobacterium tuberculosis. The blood samples did not show any significant abnormalities. The patient was given per oral clindamycin 150 mg every 8 hours and prednisolone 5 mg every 24 hours. After 8 weeks of drug treatment, decortication of mandible and additional curettage of the medullary bone were performed. The third-molar germ was also removed. Six months later, CBCT showed continued radiopacity of the mandible at the left side without any signs of active inflammation (radiolucency) (Figures 3(a) and 3(b)). Extraoral photo of patient showed a slight diffuse swelling at the left side (Figure 3(c)).

**Figure 4:**
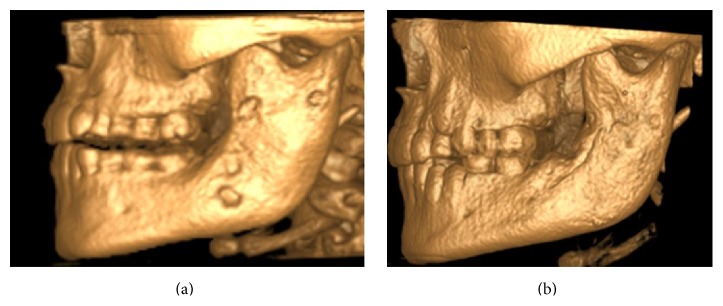
Case  1. CBCT 3D images: (a) preoperative image and (b) six-month postoperative image.

The CBCT 3D image showed continued thickness of collum mandibulae and processus coronoideus (Figures 4(a) and 4(b)). After surgery, she has received NSAID to reduce her intermittent extraoral swelling. Further investigation, treatment, and follow-up are necessary to control the patient's disease at her temporomandibular joint site.

### 5.2. Case  2

**Figure 5:**
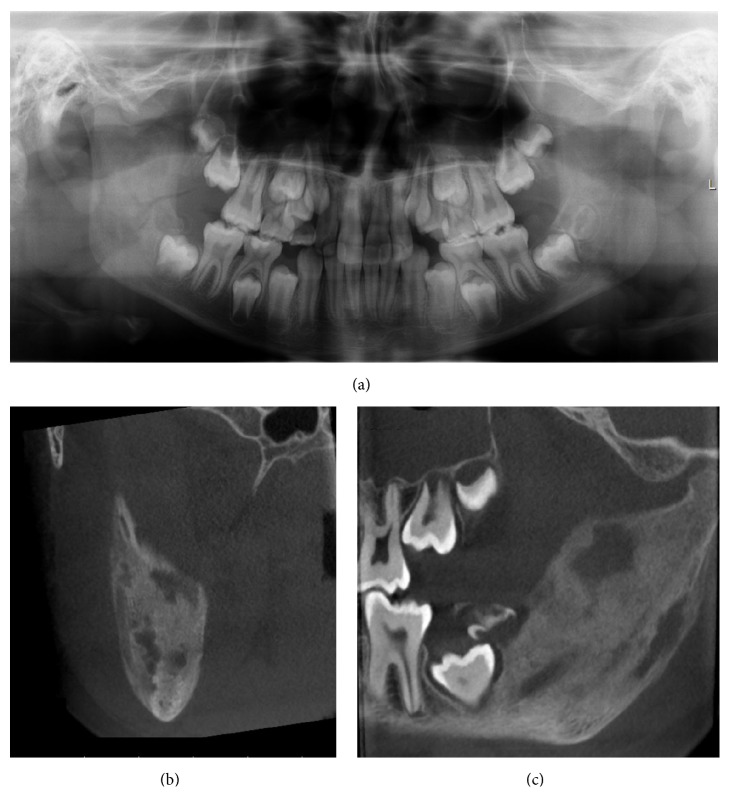
Preoperative radiographs and CBCT of patient case 2 with PCO. (a) Panoramic radiograph; (b and c) CBCT. Coronal and sagittal sections of sclerotic mandible with new bone formation and lytic lesions at the right posterior site.

An 11-year-old girl with excellent dental health was referred to the Department of Oral and Maxillofacial Surgery, Linköping University Hospital, Linköping, Sweden, for examination regarding swelling and pain on the right side of the facial skeleton. Panoramic radiograph demonstrated radiopacity within ramus/angulus mandibulae and incomplete development of third-molar germ at the right side (Figure 5(a)). Subsequent CBCT demonstrated thickness of the right lateral mandible with lytic and sclerotic lesions affecting the angle and ramus mandibulae (Figures 5(b) and 5(c)). The patient underwent diagnostic bone biopsy and the germ of third molar was removed.

**Figure 6:**
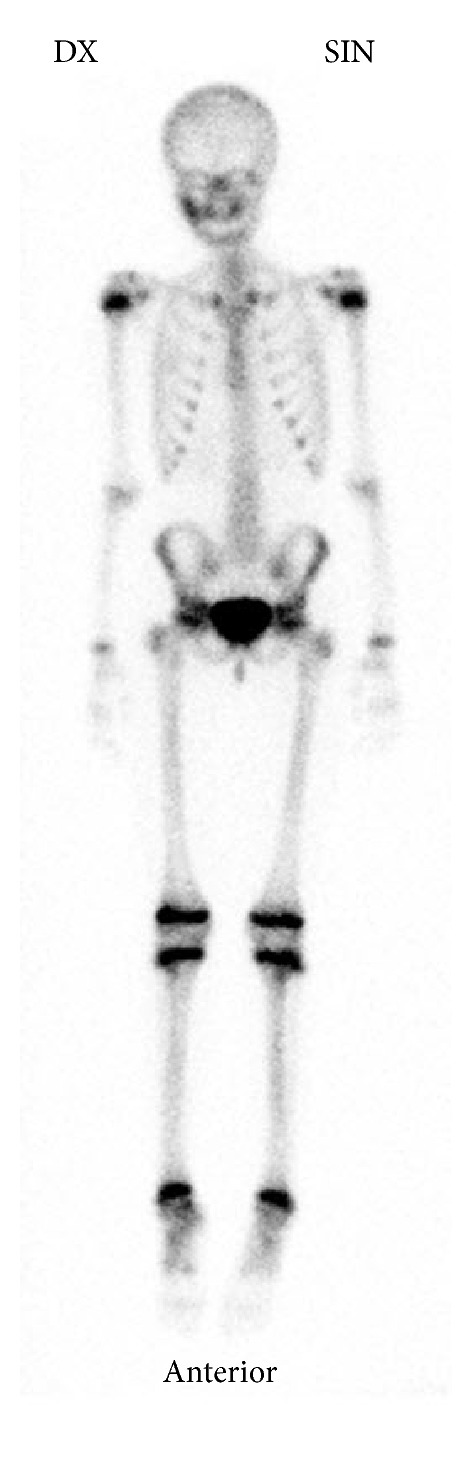
Bone scintigraphy of patient case 2 shows increased uptake of tracer at the right side of the mandible.

Histological examination predominantly showed chronic inflammatory infiltration, medullary fibrosis, and subperiosteal bone formation. No signs of malignancy were seen and the cultures for aerobic and anaerobic bacteria obtained during surgery failed to identify any bacterial growth including mycobacterium tuberculosis. The blood samples did not show any significant abnormalities. Bone scintigraphy showed increased uptake only at the right side of the mandible (Figure 6).

**Figure 7:**
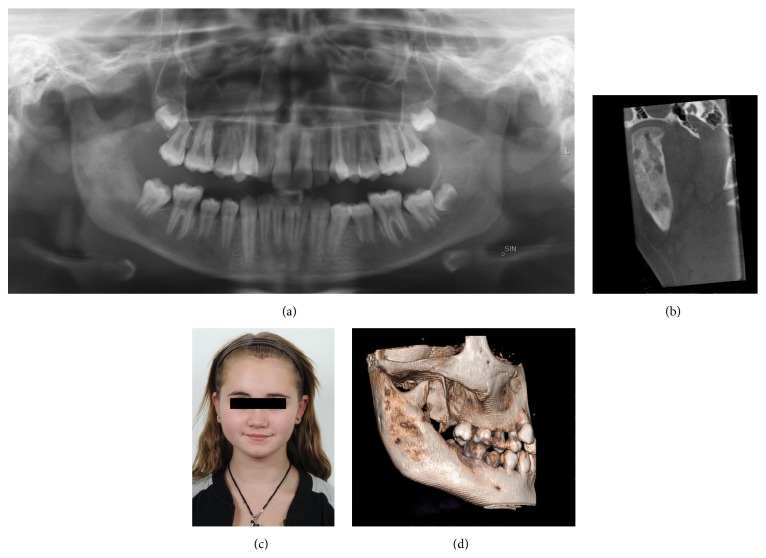
Six-month postoperative control radiographs, CBCT, and clinical image of patient case 2 with PCO. (a) Panoramic radiograph; (b) CBCT. Coronal section of the sclerotic collum mandibulae with lytic lesions; (c) patient photo, with reduced facial swelling at the right side; and (d) CT scan 3D model with bone thickness at the right side of the collum mandibulae.

Antibiotic prophylaxis was administered directly before and after bone biopsy to prevent postsurgical complications. The patient was given per oral clindamycin 150 mg every 8 hours, prednisolone 5 mg every 24 hours, and ibuprofen 600 mg every 24 hours. Full body scintigraphy showed no further involvement in the skeleton. Although the patient was on anti-inflammatory drugs, she continued to have intermittent swelling and pain. After 3 months of medication, the patient underwent decortication of mandible (corpus/ramus/angulus) and additional curettage of the medullary bone. The area around the temporomandibular joint was left without surgical intervention. Postsurgical panoramic radiographs and CBCT showed reduced subperiosteal bone thickness of mandibulae (Figures 7(a) and 7(b)). Despite this, CBCT showed signs of active inflammation (lytic lesions) within column mandibulae (Figures 7(b) and 7(c)). Patients' symptoms improved during 6 months after decortication with only a slight swelling at the right side of face being noticed (Figure 7(d)). Further controls will take place at our department, with focus on patients' temporomandibular joint area.

## 6. Results

### 6.1. Demographic, Clinical, and Histological Findings

Ten studies on PCO (purely facial skeleton involvement) were included, consisting of 3 reviews and 8 case reports (Table 1). A total number of 24 patients (6 males and 18 females) with PCO were found in the literature. The mean age of patients was 18 years (range 6–52 years) at the onset of disease. The most clinical findings were trismus, swelling, and pain [7, 14, 20, 33, 37, 65, 66]. In two cases the symptoms were preceded by lymphadenopathy (patient number 1 in our study). One patient had paresthesia of alveolar inferior nerve. The corpus and ramus mandibulae were affected in 23 patients. Four patients showed condylar involvement (including our two cases). One patient suffered from PCO located in the maxilla and zygoma. Our two cases of PCO had normal blood tests results without increase of CRP values. Histological review of patients with PCO showed chronic inflammatory infiltration, medullary fibrosis, microabscess formation, and subperiosteal bone formation [3]. The appearances of microabscess around small vessels have been reported in few patients in the literature. However this finding has not been addressed in our patients.

### 6.2. Treatment Strategies and Outcome

Additional therapeutic modalities such as antibiotics, NSAID, and corticosteroids have been reported in the literature in most of the cases (Table 2) [7, 14, 20, 33, 37, 65, 66]. Most authors preferred decortication and curettage of medullary bone in combination with anti-inflammatory drugs [7, 14, 20, 33, 65, 66]. Hyperbaric oxygen therapy, bisphosphonate, and corticosteroids injection were used in few cases [20, 65]. The mean follow-up time was 39.6 months (range 9–60 months) excluding the study by Bevin et al. [37]. The authors presented 4 cases of PCO with follow-up time of 5, 23, 26, and 34 years. Clinical symptoms (pain, swelling, and trismus) reoccurred in 7 cases. However, most of the patients with recurrence had short follow-up (less than 1 year). Our patients had significant improvement after decortication with mild remaining symptoms (slight swelling), which responded considerably well to a short course of NSAID.

## 7. Discussion

The present narrative review, based on clinical experience as well as previous reviews, indicates that PCO seems to be triggered by autoinflammatory response. The optimal treatment strategy for PCO is uncertain. Several authors advocated use of anti-inflammatory drugs as a first line of treatment [7, 66, 67]. Surgical removal of affected bone is beneficial with various outcomes [2, 14, 20, 36, 37]. PCO is characterized as a nonsuppurative chronic inflammation of the jaw bones with the absence of pus formation, extra- or intraoral fistula, or sequestration. This condition has been reported in children of both sexes with a peak onset between 10 and 20 years [8]. The most affected site is the mandible [2, 6, 7]. We presented two cases of PCO with mandibular and condyle involvement. Both patients had extraoral swelling at the affected site and trismus. Clinical examination showed full complement of teeth without any pathology or any intraoral foci of infection.

Initial suspected differential diagnoses included fibrous dysplasia, nonossifying fibroma, infection of the salivary glands, cementoma, Paget's disease, and nonspecific chronic lymphadenitis [38, 39]. Malignancy (osteosarcoma) is also included in the differential diagnosis of the PCO lesions; therefore bone biopsy is necessary for accurate diagnosis [40, 41]. Systemic blood inflammatory parameters (C-reactive protein) might be slightly elevated but usually remain within normal limits and no specific biomarkers are available for the diagnosis of these patients. One of the patients (case 1) had developed lymphadenopathy in connection with visits to the Mediterranean coast. Biopsy and blood sample were obtained to exclude osteomyelitis caused by mycobacterium tuberculosis.

Radiographic findings are characteristic, but not pathognomonic, for this condition, which presents a diagnostic challenge for oral surgeons. Several imaging modalities have been used for diagnosis of PCO, including panoramic radiograph, CT scan, CBCT scan, MRI, and skeletal scintigraphy [9–12]. A relatively recently developed acquisition technique, cone beam computed tomography (CBCT), is used in diagnosing dental, maxillofacial, and temporal bone structures [53, 54]. CBCT provides images with isotropic voxels within the range 80–400 *μ*m and gives a good overview of bone microstructure [55, 56]. Our patients' panoramic radiograph and CT scans revealed an extensive bone involvement with apparent radiolucencies and disturbed bony architecture (Figures 1(a) and 4(a)). CT scan also showed thickening of the angle/ascending ramus and periosteal reaction (Figures 1(b)-1(c) and 4(b)). The third-molar germs showed disturbed development, probably due to inflammatory process of the mandible bone. Although osteomyelitic lesions in PCO patients are mostly single, they may present multiple inflammatory focuses. Bone scintigraphy is useful in determining the presence of abnormality and the extent of disease [51, 52].

Accurate microbiological diagnosis is a cornerstone in management of osteomyelitis. Since the introduction of antibiotic therapy, the prevalence and management of osteomyelitis have been changed over the past 60 years. In PCO patients, various bacterial pathogens are reported from intraoral specimens obtained during surgery, but these were suspected to be a result of contamination [3, 31]. The mild increase in C-reactive protein and sedimentation suggests that this condition is an inflammatory process without bacterial involvement. No signs of bacterial growth have been detected in bacterial cultures from patients' blood samples. Therefore, the necessity of antibiotics in the management of PCO (despite the positive bacterial cultures) has been criticized by some authors [3, 31].

It is well known that the removal of necrotic tissue in secondary chronic osteomyelitis of the mandible has a favorable outcome [69]. However, the outcome of surgical intervention in treatment of patients with primary chronic osteomyelitis of the jaw is uncertain [2, 14, 20, 36, 37]. Surgical resection of the mandibular condyle is a possible method in adults. However, in young patients, the condyle is regarded as an important element of mandibular growth. Surgical manipulation of this region may cause a restraint of mandibular development and facial asymmetry [54]. The literature review revealed 10 studies on PCO (without syndrome involvement). Clinical symptoms reoccurred in 7 cases postoperatively and in almost all cases in whom decortication was performed. PCO is considered as an autoinflammatory disease and the removal of necrotic bone tissue as a curative therapy is still under debate.

The present study has several limitations. The content and methodology of the articles vary and therefore they are not always comparable. This condition, particularly in milder forms, is often managed by general practitioner, who might not record or publish their findings. Consequently, this would result in an underestimation of the number of PCO cases and would therefore hamper an accurate calculation of the true number of cases.

## 8. Conclusion

PCO is nonsuppurative chronic inflammatory disorder in which bone is the primary inflammatory target. Surgical removal of affected bone is beneficial in some cases. In patients with mandible condyle involvement, anti-inflammatory drugs such as NSAID and corticosteroids seem to be the first-line options.

## Figures and Tables

**Table 1:** Demographic and clinical outcome.

Studies PCO	No	Symptoms	Site of origin (number)	Gender M/F	Age of onset Mean/range
Heggie et al., 2003 [33]	2	Pain, swelling	Unilateral mandibular enlargement (**2**)	0M/8F	10,5/7–12
Yamazaki et al., 2007 [65]	1	Pain, swelling, and trismus	Corpus-ramus mandibulae (**1**)	1M/0F	9
Lentrodt et al., 2007 [20]	3	Pain, swelling	Hemimandible (**3**)	1M/2F	11,5/10–13
Bevin et al., 2008 [37]	4	Pain, swelling	Hemimandible (**4**)	0M/4F	25/13–52
Theologie-Lygidakis et al., 2011 [14]	5	Pain, swelling, trismus, and lymphadenopathy	Angulus-ramus mandibulae (**2**) Corpus-angulus mandibulae (**2**) Maxilla and zygoma complex (**1**)	1M/4F	7/3–9
Obel et al., 2013 [36]	3	Pain, swelling, and trismus	Hemimandible (**3**)	1M/2F	9/6–11
Ventin and Eguido, 2006 [67]	1	Pain, erythema of skin, and paresthesia of alveolar inferior nerve	Hemimandible (**1**)	1M/0F	23
Agarwal et al., 2014 [7]	1	Pain, swelling, and paresthesia of alveolar inferior nerve	Corpus mandibulae (**1**)	0M/1F	28
Kanemoto et al., 1992 [68]	1	Pain, swelling, and trismus	Condyle mandibulae (**1**)	1M/0F	14
Zemann et al., 2011 [66]	1	Pain, swelling, and trismus	Condyle mandibulae (**1**)	0M/1F	51
Berglund, 2015 (The present study)	2	Pain, swelling, and trismus Lymphadenopathy	Corpus-condyle (**1**) Angulus-condyle (**1**)	0M/2F	11,5/11-12
Total	24			6M/18F	Mean 18.1 years

**Table 2:** Treatment strategies and outcome.

Studies	Drug therapy	Surgical treatment	Other treatments	Follow-up time	Number/CR
Heggie et al., 2003 [33]	AB, NSAID	Decortication	No	60	2/1
Yamazaki et al., 2007 [65]	AB, NSAID	Decortication	Pamidronate HBOT	60	1/1
Lentrodt et al., 2007 [20]	AB	Decortication, removal of third-molar germ	HBOT	42	3/0
Bevin et al., 2008 [37]	AB	Extraction of teeth, partial resection of mandible	No	264 (60–408)	4/1
Theologie-Lygidakis et al., 2011 [14]	AB, NSAID	Decortication	No	24	5/2
Obel et al., 2013 [36]	AB, NSAID, P	No	Triamcinolone injection	51	3/0
Ventin and Eguido, 2006 [67]	AB	No	No	24	1/0
Agarwal et al., 2014 [7]	AB	Decortication	No	24	1/0
Kanemoto et al., 1992 [68]	AB	Decortication	Cefmetazole sodium Fosfomycin sodium	54	1/0
Zemann et al., 2011 [66]	AB	No	No	48	1/0
Berglund, 2015 (The present study)	AB, NSAID, P	Decortication, removal of third-molar germ	No	9	2/2

AB = antibiotic, NSAID = nonsteroidal anti-inflammatory drug, P = prednisolone, and CR = clinical recurrence.
